# Unlike dietary restriction, rapamycin fails to extend lifespan and reduce transcription stress in progeroid DNA repair‐deficient mice

**DOI:** 10.1111/acel.13302

**Published:** 2021-01-23

**Authors:** María B. Birkisdóttir, Dick Jaarsma, Renata M. C. Brandt, Sander Barnhoorn, Nicole van Vliet, Sandra Imholz, Conny T. van Oostrom, Bhawani Nagarajah, Eliana Portilla Fernández, Anton J. M. Roks, Ype Elgersma, Harry van Steeg, José A. Ferreira, Jeroen L. A. Pennings, Jan H. J. Hoeijmakers, Wilbert P. Vermeij, Martijn E. T. Dollé

**Affiliations:** ^1^ Princess Máxima Center for Pediatric Oncology, Genome Instability and Nutrition ONCODE Institute Utrecht The Netherlands; ^2^ Department of Neuroscience Erasmus MC Rotterdam The Netherlands; ^3^ Department of Molecular Genetics Erasmus MC Rotterdam The Netherlands; ^4^ Centre for Health Protection National Institute for Public Health and the Environment (RIVM Bilthoven The Netherlands; ^5^ Division of Vascular Medicine and Pharmacology Department of Internal Medicine Erasmus MC Rotterdam The Netherlands; ^6^ Department of Statistics, Informatics and Modelling National Institute for Public Health and the Environment (RIVM Bilthoven The Netherlands; ^7^ CECAD Forschungszentrum Köln Germany

**Keywords:** aging, dietary restriction, DNA damage repair, rapamycin, transcription stress

## Abstract

Dietary restriction (DR) and rapamycin extend healthspan and life span across multiple species. We have recently shown that DR in progeroid DNA repair‐deficient mice dramatically extended healthspan and trippled life span. Here, we show that rapamycin, while significantly lowering mTOR signaling, failed to improve life span nor healthspan of DNA repair‐deficient *Ercc1* ^∆/−^ mice, contrary to DR tested in parallel. Rapamycin interventions focusing on dosage, gender, and timing all were unable to alter life span. Even genetically modifying mTOR signaling failed to increase life span of DNA repair‐deficient mice. The absence of effects by rapamycin on P53 in brain and transcription stress in liver is in sharp contrast with results obtained by DR, and appoints reducing DNA damage and transcription stress as an important mode of action of DR, lacking by rapamycin. Together, this indicates that mTOR inhibition does not mediate the beneficial effects of DR in progeroid mice, revealing that DR and rapamycin strongly differ in their modes of action.

## Graphical abstract

In this study, we have subjected *Ercc1* ^Δ/−^ progeroid DNA repair‐deficient mice to rapamycin and dietary restriction (DR), two well‐known interventions for extending life span across multiple species. DR again resulted in extreme benefits for these mice while rapamycin interventions, focusing on dosage, gender, and timing as well as modulation of mTor via genetic means, all failed to improve life span, healthspan, or the DNA damage indicator transcription stress.

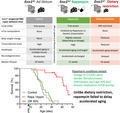

## 1 INTRODUCTION

Caloric or dietary restriction (CR/DR; reduced total dietary intake without malnutrition) is the best documented intervention that compresses morbidity and extends life span (Fontana et al., 2010; Speakman & Mitchell, 2011). Restricting diet under laboratory conditions has been shown to promote health and delay aging in numerous species ranging from yeast to non‐human primates, suggesting an evolutionary well‐conserved mechanism underlying the life span‐extending effect of DR (Fontana & Partridge, 2015; Fontana et al., 2010; Mair & Dillin, 2008). Human DR studies have also shown beneficial health effects (Kraus et al., 2019; van de Rest et al., 2016) but controlled studies on long‐term consequences of DR in humans are lacking. Moreover, it remains to be seen how humans would react to an extended period of DR in terms of compliance and potential side effects of DR (Dirks & Leeuwenburgh, 2006; Most et al., 2017).

Despite decade‐long research, the mechanisms underlying DR are still poorly understood, although energy expenditure with suppression of nutrient‐sensing pathways via GH/IGF1 and mTOR signaling has been reported as two primary regulatory pathways in the healthspan and life span extension by DR (Barzilai et al., 2012; Finkel, 2015; Liu & Sabatini, 2020; Lopez‐Otin et al., 2016; Speakman & Mitchell, 2011). To find alternatives to DR, studies have focused on CR/DR mimetics: compounds that mimic DR by targeting anti‐aging mechanisms induced by DR, such as metabolic and stress response pathways, but without actually restricting dietary intake, avoiding potential downsides of DR: for example, constant hunger feeling (Ingram & Roth, 2015). To date, the most robust intervention, besides DR, is rapamycin (Blagosklonny, 2019; Fontana et al., 2010; Ingram & Roth, 2015). Rapamycin, also known as sirolimus, reduces the nutrient‐sensing mTOR pathway, and was suggested a DR mimetic early 2000 (Sharp & Bartke, 2005). Rapamycin, as well as genetic modulation of the serine/threonine protein kinase mTOR, has consistently been found to extend life span in numerous species (Barbet et al., 1996; Garratt et al., 2016; Kaeberlein et al., 2005; Kapahi et al., 2004; Savage, 2017; Urfer et al., 2017; Vellai et al., 2003). In mice, overall, life span was modestly extended, most prominently in females (Garratt et al., 2016; Harrison et al., 2009; Miller et al., 2014; Selman et al., 2009).

We have previously reported (Vermeij, Dollé, et al., 2016) that—counterintuitively—severely growth‐attenuated, progeroid DNA repair mutant mice benefit extraordinarily from DR: Whereas 30% DR in wild‐type (WT) mice leads on average to a 30% increase in life span (Weindruch & Sohal, 1997), the increase in life span of short‐lived *Ercc1* ^∆/−^ progeroid mice was more than 2‐fold (Vermeij, Dollé, et al., 2016). As *Ercc1* ^∆/−^ mutant mice are defective in multiple DNA repair processes including transcription‐coupled repair (TCR), global‐genome nucleotide excision repair (GG‐NER), and interstrand crosslink repair, several types of endogenous DNA lesions, which are normally repaired by these processes, accumulate faster over time (Marteijn et al., 2014; Vermeij et al., 2014). As a consequence of increased DNA damage, one of the hallmarks of aging (Lopez‐Otin et al., 2013), *Ercc1* ^∆/−^ mice, and corresponding human patients with progeroid DNA repair deficiency syndromes, shows a broad spectrum of accelerated aging phenotypes. For *Ercc1* ^∆/−^ mice, life span is reduced to about 4–6 months during which they develop widespread multimorbidity also seen in normal mouse and human aging (Dollé et al., 2011; Vermeij et al., 2016). Applying DR to these and other progeroid DNA repair mutant mice, dramatically extended life span, improved overall health and delayed numerous aspects of aging, including neurodegeneration. More importantly, it reduced the endogenous DNA damage load and transcription stress, lowering one of the causal hallmarks of aging (Vermeij, Dollé, et al., 2016; Vermeij, Hoeijmakers, et al., 2016). Hence, progeroid DNA repair mutant mice appear a well‐suited model for examining potential DR mimetics like rapamycin (Ingram & Roth, 2015).

## 2 RESULTS

### 2.1 **Effect of rapamycin on life span of *Ercc1*** ^∆^ **^/−^ mice**

**FIGURE 1:**
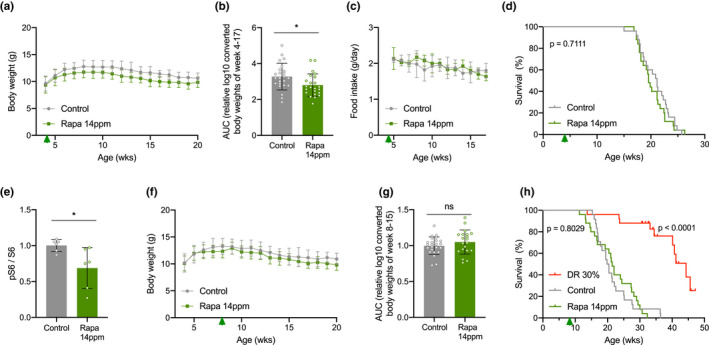
Different timing regimes of rapamycin feeding do not extend life span of *Ercc1* ^∆/−^ mouse mutants. a, Mean body weights development (±SD) of female mice with *ad libitum* access to control AIN93G diet (gray) or 14 ppm rapamycin supplemented diet (green) provided from 4 weeks of age (arrowhead). b, Body weights as area under the curve (AUC) for each condition (for details, see Table S1). c‐d, Corresponding mean food intake (±SD) (c) and life span (d) curves. *n* = 25 animals per group. e, Quantified relative S6 phosphorylation by rapamycin in *Ercc1* ^∆/−^ liver extracts. Six animals per group were used treated from 8 to 11 weeks of age. Mean ± SD and individual datapoints are indicated. *p < 0.05. f–h, Body weight (f) and AUC thereof (for details, see Table S1) (g) (both mean ± SD) and survival curves (h) of a second cohort of female *Ercc1* ^∆/−^ mice fed 14 ppm rapamycin diet (green) or 30% dietary restriction (DR; red) from 8 weeks of age (arrowhead). *n* = 24 animals per group for control and *n* = 25 for rapamycin and DR (15 censored for survival but indicated as currently still alive). *p* values of the log‐rank survival test are indicated and refer to the comparison of each intervention group to its own control. The direct comparison of DR versus rapamycin yielded a *p* < 0.001

Rapamycin in an encapsulated form was added to the mouse feed, at a dosage of 14 mg/kg food (14 ppm), conditions previously shown effective for WT mice (Harrison et al., 2009). Expressed per gram body weight *Ercc1* ^∆/−^ mice eat 1.3 times more than their repair‐proficient siblings (Dollé et al., 2011), indicating that the oral dose of rapamycin per gram body weight is slightly higher than in experiments with WT mice. As *Ercc1* ^∆/−^ progeroid DNA repair mutant mice age accelerated with a median life span of about 20 weeks (Dollé et al., 2011; Vermeij, Hoeijmakers, et al., 2016), we applied rapamycin early in life, from 4 weeks of age onwards, that is, directly at weaning. At this age, *Ercc1* ^∆/−^ progeroid mice still show minor indications (e.g., growth retardation) of DNA damage accumulation (de Waard et al., 2010; Dollé et al., 2011; Vermeij, Hoeijmakers, et al., 2016). We first focused on females as in previous studies they showed the biggest improvement following rapamycin treatment (Garratt et al., 2016; Miller et al., 2014). We monitored body weight and food intake to examine whether addition of rapamycin to the food did not artificially induce a DR response. As observed by others (Miller et al., 2011), rapamycin feeding lowered overall body weight by about 5%–10% (Figure 1a,b and Table S1). Importantly, rapamycin‐fed mice did not show reduced food intake (Figure 1c), excluding indirect effects of DR for which *Ercc1* ^∆/−^ mice are very sensitive (Vermeij, Dollé, et al., 2016). However, when monitoring survival of *Ercc1* ^∆/−^ mice fed *ad libitum* control or rapamycin diet, we failed to find any increase in median or maximum life span (Figure 1d).

One possible explanation is that rapamycin was administered too early in development. Moreover, rapamycin appears to work predominantly later in life (Harrison et al., 2009). Therefore, we repeated the study in a second cohort of *Ercc1* ^∆/−^ females, now starting the rapamycin diet at 8 weeks of age, that is, early adulthood. At this age, *Ercc1* ^∆/−^ progeroid mice have developed first clear symptoms of aging (de Waard et al., 2010; Dollé et al., 2011; Vermeij, Hoeijmakers, et al., 2016), and at this age, we had previously successfully applied DR leading to ~200% life span extension (Vermeij, Dollé, et al., 2016). This time, we first tested the effectiveness of nutritional supplementation of 14 ppm rapamycin on mTORC1 activity in *Ercc1* ^∆/−^ mice by measuring phosphorylation of ribosomal protein S6 (at Serine^240^ and Serine^244^), and we took dietary restriction along as a positive control. Rapamycin indeed reduced S6^S240/244^ phosphorylation by about one‐third analyzed after three weeks compared to mock‐treated controls (Figure 1e and Figure S1). We did not notice significant suppression of body weight or food intake, in contrast with DR (Figure 1f,g, Figure S2a–c and Table S1). While 30% DR confirmed the impressive life span extension in a new cohort of *Ercc1* ^∆/−^ females, rapamycin failed to improve median or maximal life span (Figure 1h). *Ercc1* ^∆/−^ mice subjected to 30% DR lived significantly longer than each of the AL control (*p* = 3,2 × 10^−10^) and rapamycin‐fed (*p* = 6,1 × 10^−11^) mice.

**FIGURE 2:**
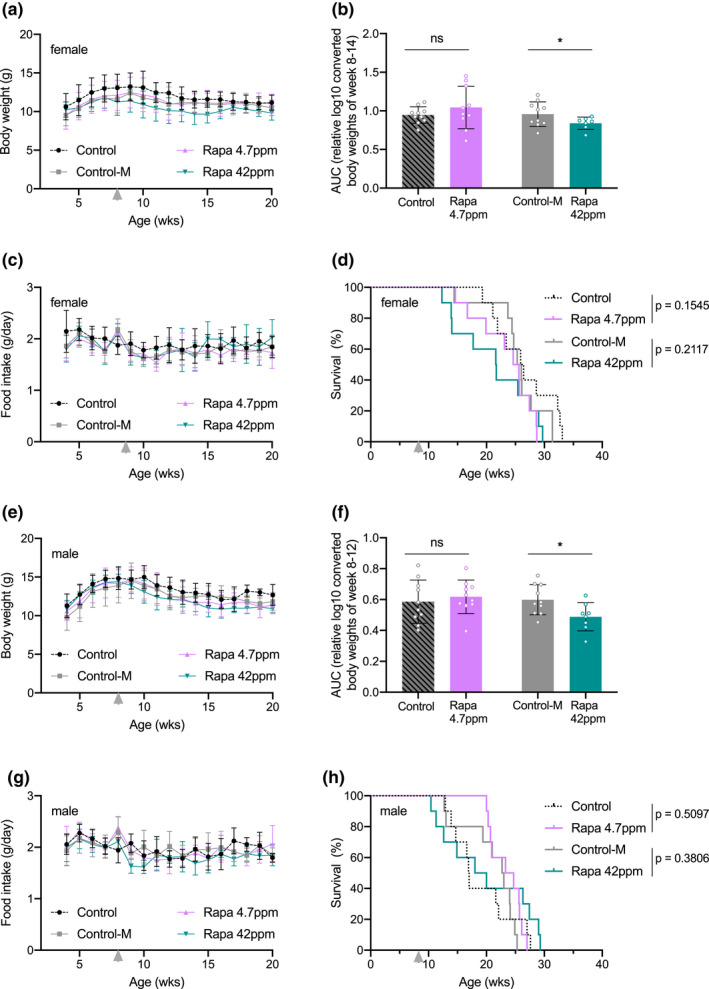
Altering dosage of rapamycin does not extend life span of *Ercc1* ^∆/−^ mouse mutants in both genders. Body weight means per week (a, e), body weight AUC (for details see Table S1) (b, f), food intake (c, g), and survival (d, h) curves of female (a–d) and male (e–h) *Ercc1* ^∆/−^ mice fed *ad libitum* control AIN93G diet (black dotted lines; Control), supplemented with empty microcapsules (gray; Control‐M), 4.7 ppm rapamycin (pink), or 42 ppm rapamycin (blue) from 8 weeks of age (arrowhead). *p* values of the log‐rank survival test are indicated and refer to the comparison of each intervention group to its own control. *n* = 10 animals per group. Error bars denote mean ± SD *p < 0.05

To examine whether *Ercc1* ^∆/−^ mutants require a different dose of rapamycin, or to identify any potential toxic effect in these mice, we started other cohorts with three times higher (42 ppm) and lower (4.7 ppm) dosages of dietary rapamycin. Alongside the unsupplemented control group (Control), we now also used a control group supplemented with the empty encapsulation material (Control‐M), equivalent to that used in the highest rapamycin dose. In addition, we also included male mice to control for potential gender specific effects. The treatment with rapamycin reduced body weight, in a dose‐dependent manner (Figure 2a,b,e,f and Table S1), without affecting food intake (Figure 2c,g). However, altered doses of rapamycin still failed to extend (or shorten) life span of *Ercc1* ^∆/−^ mice (Figure 2d,h), in line with the 14 ppm treatment.

### 2.2 Effect of rapamycin on parameters of healthspan of *Ercc1^Δ^* ^/−^ mice

Although rapamycin appears inert in extending life span it might still alleviate some of the features of organ functional decline that were previously rescued by DR (Vermeij, Dollé, et al., 2016). Rapamycin, like DR, is known for reducing cancer (Anisimov et al., 2010; Blagosklonny, 2012; Livi et al., 2013; Rao et al., 2004). Neoplastic lesions were not observed in any of the moribund and cross‐sectional animals fed rapamycin or control diet, consistent with the previous observation of absent neoplasia in short‐lived *Ercc1* ^∆/−^ mice (Dollé et al., 2011). Additionally, no obvious macroscopical differences in major organs and tissues were noted between rapamycin‐fed and control‐treated animals.

The liver exhibits substantial age‐related pathology in *Ercc1* ^∆/−^ mice (Dollé et al., 2011; Gregg et al., 2012; Selfridge et al., 2001), in particular hepatocyte polyploidization that possibly follows from unrepaired DNA interstrand cross‐links (Grillari et al., 2007) and is strongly reduced by DR (Vermeij, Dollé, et al., 2016). Flow cytometry confirmed the rise in the fraction of large 16 N polyploid nuclei in the aging *Ercc1* ^∆/−^ liver, but unlike DR, medium‐dose rapamycin (14 ppm) feeding did not attenuate the increase in polyploidization (Figure S2d).

To explore effectiveness of high‐dose rapamycin (42 ppm), we examined its impact on endothelial function by studying aortic endothelium‐dependent relaxation to acetylcholine (ACh) in organ bath setups. Unlike DR, which is consistently protective, rapamycin has been reported as toxic in human aged arteries and in rodent models, but paradoxically also protective in blood vessels in healthy aging mice (Habib et al., 2014; Harari et al., 2018; Jabs et al., 2008; Kim et al., 2010; Lesniewski et al., 2017). Contrary to previously reported protection by DR (Vermeij, Dollé, et al., 2016; Wu et al., 2017), endothelium‐dependent responses were decreased by rapamycin in both *Ercc1* ^∆/−^ and WT mice, supporting the toxic effects that were reported, among others, in human aged arteries (Figure S3a,b).

**FIGURE 3:**
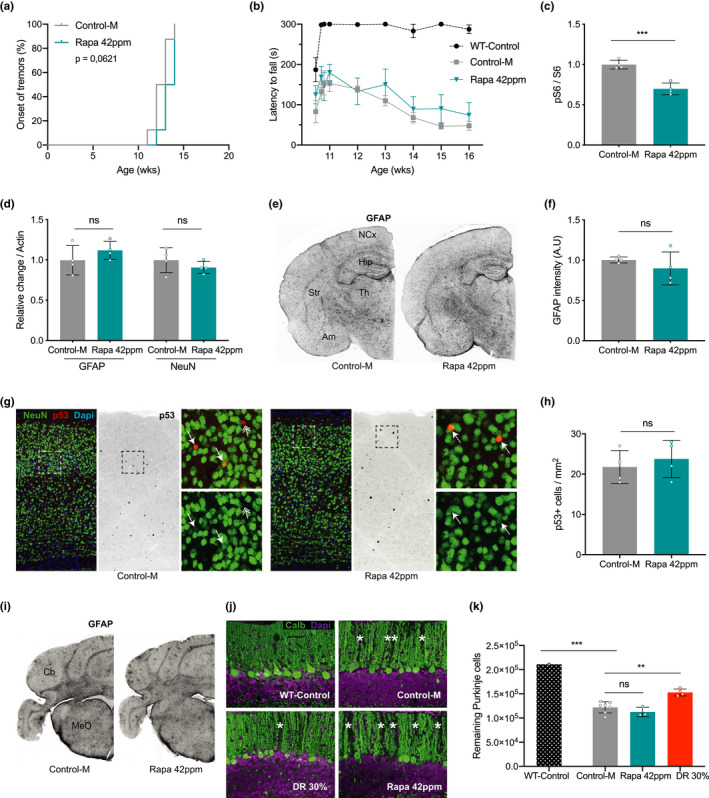
Rapamycin does not prevent nervous system abnormalities in *Ercc1* ^∆/−^ mice. a, Onset of the neurological abnormality tremors with age in *ad libitum* control AIN93G diet supplemented with empty microcapsules (gray; Control‐M) and 42 ppm rapamycin (blue) fed *Ercc1* ^∆/−^ mice. *n* = 8 animals per group. b, Average time spent on an accelerating rotarod of WT and *Ercc1* ^∆/−^ mice weekly monitored between 11 and 16 weeks (*n* = 4; WT *n* = 3). A daily training period was given at 10 weeks of age. c, d, Quantified relative S6 phosphorylation (c) and levels of GFAP (astrocytosis) and NeuN (neurons) (d) by rapamycin in *Ercc1* ^∆/−^ neocortex extracts. Four animals per group were used treated from 8 to 16 weeks of age. e, Representative pictures of transverse brain sections of 16‐week‐old control and rapamycin‐fed *Ercc1* ^∆/−^ mice IF‐stained for GFAP. f, Quantification of the relative intensity of consecutive transverse brain sections immunoperoxidase‐stained for GFAP in neocortex. g, Representative immunofluorescent confocal images of the neocortex stained for NeuN (green) and p53 (red). h, p53‐positive cells counted in the neocortex of three consecutive transverse brain sections at the level of the bregma (Mouse Brain Atlas, Paxinos). i, Representative pictures of cerebellar brain sections of 16‐week‐old control and rapamycin‐fed *Ercc1* ^∆/−^ mice IF‐stained for GFAP. j, Representative immunofluorescent confocal images of the cerebellum stained for Purkinje neurons by Calbindin (green). k, Stereological quantification of the total number of Purkinje neurons per brain. *n* = 3–6 mice. Mean ± SD and individual datapoints are indicated. **p < 0.01, ***p < 0.001

*Ercc1* ^∆/−^ mice develop progressive neurological and neurodegenerative changes (Borgesius et al., 2011; de Waard et al., 2010; Jaarsma et al., 2013), which are strongly attenuated by DR (Vermeij, Dollé, et al., 2016). Next, we investigated the effect of high‐dose rapamycin (42 ppm) on neurological and neurodegenerative changes in *Ercc1* ^∆/−^ mice. This cohort was examined weekly for the onset of tremors and loss of motor performance on an accelerating rotarod; two neurological symptoms that develop in a predictable way in *Ercc1* ^∆/−^ mice (de Waard et al., 2010) and that are dramatically delayed or attenuated in DR‐treated animals (Vermeij, Dollé, et al., 2016). Rapamycin did not alter the onset of tremors (Figure 3a), nor improve accelerating rotarod performance (Figure 3b and Figure S3c). At 16 weeks of age, when showing severe motor abnormalities, the mice were sacrificed for neuropathological and biochemical analyses. Immunoblot analysis confirmed reduced relative S6^S240/244^ phosphorylation (Figure 3c and Figure S4a,b) in neocortex, consistent with mTORC1 inhibition and liver data (Figure 1e). In line with mTORC1 inhibition, we also observed a trend, albeit non‐significant, of increased expression of markers of autophagy (Figure S3d,e and Figure S4c,d).

Using immunoblots, we next examined proxies of neuronal injury using GFAP, a marker that correlates with reactive astrocytosis, and NeuN, a general neuronal marker (Borgesius et al., 2011; de Waard et al., 2010). Rapamycin‐ and control‐treated *Ercc1* ^∆/−^ mice expressed similar levels of GFAP and NeuN, suggestive of equal levels of neuronal injury (Figure 3d and Figure S4e,f). Immunohistology confirmed that rapamycin‐fed mice displayed the same levels of GFAP immunoreactivity in the neocortex (Figure 3e,f). In addition, we found that the density of P53‐positive nuclei, representing a marker of genotoxic stress, is unaltered by rapamycin treatment in the neocortex (Figure 3g,h, arrows). In summary, our results show that rapamycin has no effect on behavioral deficits or neuropathology in *Ercc1* ^∆/−^ mice, whereas these parameters were previously largely rescued by DR (Vermeij, Dollé, et al., 2016).

To further explore the differences between DR and rapamycin treatment in attenuating nervous system abnormalities in *Ercc1* ^∆/−^ mice, we examined the cerebellum for GFAP staining (Figure 3i) and we directly compared cerebellar Purkinje cell degeneration from the current rapamycin and previously collected DR cohorts (Vermeij, Dollé, et al., 2016). Purkinje cells display relatively high vulnerability in aging humans and rodents (Andersen et al., 2003; Woodruff‐Pak et al., 2010), as well as progeroid DNA repair deficiency patients (Jaarsma et al., 2011, 2013; Weidenheim et al., 2009), and their degeneration may underlie motor dysfunction in progeria patients and mouse models (Jaarsma et al., 2013; Weidenheim et al., 2009). Immunostaining for calbindin to selectively outline Purkinje cells revealed multiple gaps in the staining of the molecular layer of control and rapamycin‐treated *Ercc1* ^∆/−^ mice, indicative of Purkinje cell loss, while in DR cerebellum the PCs appeared more preserved (Figure 3j, asterisks). Importantly, quantitative analysis of Purkinje cells using a stereological approach confirmed that rapamycin‐fed *Ercc1* ^∆/−^ mice showed a similar degree of Purkinje cell loss as *ad libitum* control fed *Ercc1* ^∆/−^ mice, while Purkinje cell loss was significantly attenuated by DR (Figure 3k). Taken together, our results reveal that rapamycin, unlike DR, does not extend healthspan and is not neuroprotective in progeroid *Ercc1* ^∆/−^ mice.

### 2.3 Genetically altering mTOR activity does not influence life span of *Ercc1* ^∆^^/−^ mice

The lack of effect of rapamycin indicates that inhibition of mTOR signaling does not interfere with life span of *Ercc1* ^∆/−^ mice and that mTOR inhibition does not mediate the beneficial effect of DR in these mice. However, it is not excluded that in our DNA repair mutant mice rapamycin exerts additional negative effects other than via inhibiting mTOR activity. Therefore, we investigated modulation of mTOR activity bidirectionally via a genetic approach using *Rheb* ^+/−^ mice (Goorden et al., 2011) and *Tsc1* ^+/−^ mice (Wilson et al., 2005), two key upstream regulators of mTORC1: RHEB is a small Ras‐related GTPase that stimulates mTORC1 activity, while TSC1 is a GTPase‐activating protein that converts RHEB to its GDP‐bound state thereby inactivating mTORC1 activity (Liu & Sabatini, 2020). As both mouse mutations are homozygously lethal, heterozygously mutating *Rheb* or *Tsc1 *has been shown to lower protein expression, and respectively suppress or enhance mTORC1 activity by one third (Goorden et al., 2011; Wesseling et al., 2017). Both these mouse models, which were studied previously in light of neuronal development but never life span, were crossed with *Ercc1* ^∆/−^ mice in the same F1 C57BL6J/FVB hybrid background. In addition, we also crossed both lines with *Xpg* ^−/−^ mice, an additional DNA repair deficiency mouse model, with a highly similar life span and accelerated aging phenotype, that could also be substantially delayed by DR (Barnhoorn et al., 2014; Vermeij, Dollé, et al., 2016).

**FIGURE 4:**
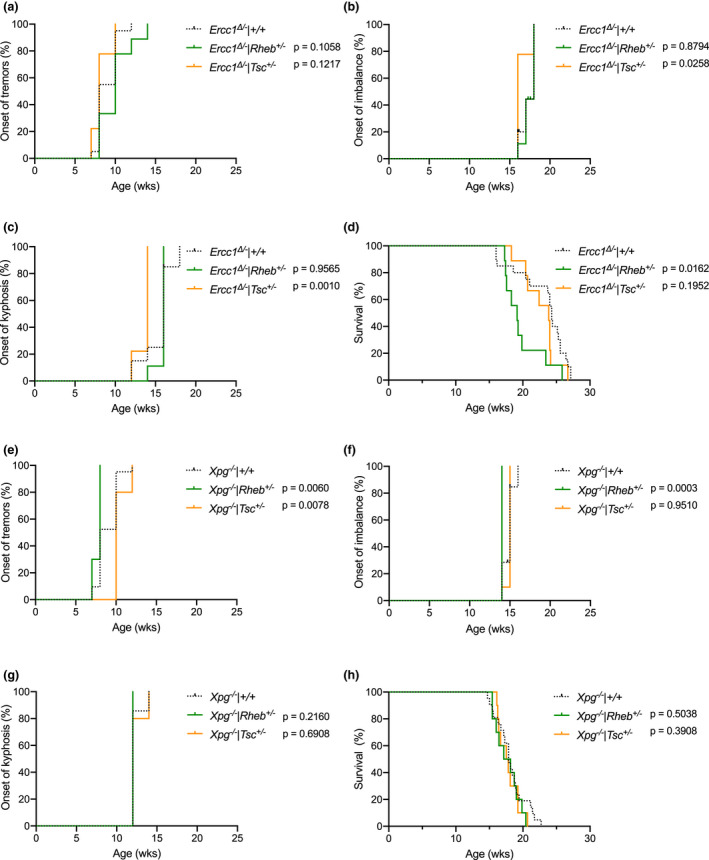
Genetically altering mTOR activity does not influence life span of *Ercc1* ^∆/−^ mice. a–c, Onset of neurological abnormalities, tremors (a) and imbalance (b), and kyphosis (c) with age in *Ad libitum* fed *Ercc1* ^∆/−^ *Rheb* ^+/−^ (green lines; reduced mTOR) and *Ercc1* ^∆/−^ *Tsc* ^+/−^ (orange lines; increased mTOR) mutant mice and littermate control *Ercc1* ^∆/−^ mice (black dotted lines). d, Survival of *Ercc1* ^∆/−^ *Rheb* ^+/−^ (green lines; reduced mTOR) and *Ercc1* ^∆/−^ *Tsc* ^+/−^ (orange lines; increased mTOR) mutant mice and littermate control *Ercc1* ^∆/−^ mice (black dotted lines). *n* = 9 animals per group for *Rheb* ^+/−^ and *Tsc* ^+/−^ mutants and 20 for *Ercc1* ^∆/−^ controls. e–h, survival curves (e) and onset of neurological abnormalities, tremors (f) and imbalance (g), and kyphosis (h) with age in *Ad libitum* fed *Xpg* ^−/−^ *Rheb* ^+/−^ (green lines; reduced mTOR) and *Xpg* ^−/−^ *Tsc* ^+/−^ (orange lines; increased mTOR) mutant mice and littermate control *Xpg* ^−/−^ mice (black dotted lines). *n* = 10 animals per group for *Rheb* ^+/−^ and *Tsc* ^+/−^ mutants and 21 for *Xpg* ^−/−^ controls. *p* values of the log‐rank test are indicated and refer to the comparison of each intervention group to its own control. When stratified for gender, log‐rank survival estimate *p* values were 0.0843 and 0.1983 for respectively *Rheb* ^+/−^ and *Tsc* ^+/−^ mutants in the *Ercc1* ^∆/−^ background, and 0.4888 and 0.2631 for respectively *Rheb* ^+/−^ and *Tsc* ^+/−^ mutants in the *Xpg* ^−/−^ background

Consistent with the rapamycin treatment, reduced mTORC1 activity resulting from deletion of one *Rheb* allele did not alter the time of onset of neurological deficits (tremors and imbalance), the time of onset of kyphosis, and life span in *Ercc1* ^∆/−^ mice (Figure 4a‐d; green lines) nor in *Xpg* ^−/−^ mice (Figure 4e‐h; green lines). Similarly, both *Ercc1* ^∆/−^ and *Xpg* ^−/−^ mice crossed with *Tsc1* ^+/−^ mice, experiencing chronic upregulation of mTORC1 activity, showed unaltered life span and time of onset of aging‐related phenotypes (Figure 4 and Figure S5). These findings indicate that also by influencing mTORC1 activity genetically, our DNA repair‐deficient prematurely aging mice appear insensitive to the healthspan and life span‐modulating activity of mTOR inhibition.

### 2.4 Effect of rapamycin on age‐related transcription stress

**FIGURE 5:**
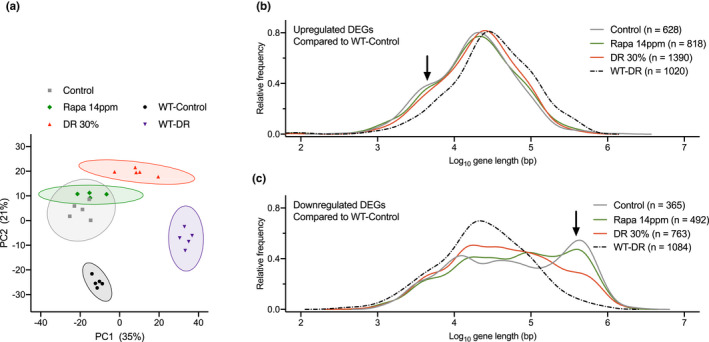
Rapamycin does not prevent transcription stress in *Ercc1* ^∆/−^ liver. a, Principal component analysis (PCA) of full genome liver RNA expression profiles of 11‐week‐old *Ercc1* ^∆/−^ mice fed *ad libitum* control diet (gray squares), diet supplemented with 14 ppm rapamycin (green diamonds), or fed 30% DR (red triangles). WT mice were fed *ad libitum* control diet (black circles) or 30% DR (purple triangles). This analysis takes into account all the genes in the microarray platform. The two main principal components, PC1 and PC2, explain 56% of the variability in the original dataset: PC1 (*x*‐axis, 35%) differentiates on the basis of expression changes induced by DR, independent of genotype; PC2 (*y*‐axis, 21%) reflects differences associated with genotype. 95% confidence intervals are indicated by colored circles around the dots. b, c, Relative frequency plot of gene length (log scale) of DEGs in 11‐week‐old WT mice given 30% DR (WT‐DR) versus *ad libitum* WT‐Control (black dashed lines), *Ercc1* ^∆/−^ mice fed Control diet versus WT‐Control (gray lines), *Ercc1* ^∆/−^ mice given 30% DR versus WT‐Control (red lines), and *Ercc1* ^∆/−^ mice fed 14 ppm rapamycin diet versus WT‐Control (green lines). b, Only upregulated genes. c, Only downregulated genes. Black arrows, extra peak of upregulated short genes (b) and peak of downregulated long genes (c) in both *Ercc1* ^∆/−^ Control and Rapa 14 ppm mice. Average gene lengths and *p* values of comparisons are indicated in Table S3. *n* = 4–5 animals per group

To obtain a more mechanistic understanding of the large difference in effects of rapamycin treatment vs DR on survival of *Ercc1* ^∆/−^ mice, we next examined the effect of 14 ppm rapamycin on the liver full genome transcriptome of *Ercc1* ^∆/−^ mice versus WT controls and compared it to simultaneously re‐analyzed changes induced by DR. Unbiased principal component analysis revealed high similarity between *Ercc1* ^∆/−^ mice fed *ad libitum* control (Control) or rapamycin (Rapa 14 ppm) diet (Figure 5a and Figure S6a), whereas *Ercc1* ^∆/−^ mice given a restricted diet (DR 30%) were clearly separated, like WT mice fed *ad libitum* control diet (WT‐Control) or subjected to 30% dietary restriction (WT‐DR) (Figure 5a and (Vermeij, Dollé, et al., 2016). As microarray expression revealed no significant gene expression changes between *Ercc1* ^∆/−^ mice on rapamycin or control diet (using FDR < 0.05 with FC ≥ |1.5|), consistent with PCA analysis, we decided to compare both groups to WT’s fed control diet, again showing consistent overlapping Gene Ontology pathways and upstream regulator transcription factors (Table S2). Likewise, at 11 weeks of age we did notice some DNA damage response and cell fate markers similarly affected in AL control and rapamycin‐fed *Ercc1* ^∆/−^ mice, while differing upon DR treatment: for example, CDKN1A expression was somewhat elevated in livers of *Ercc1* ^∆/−^ mice (control and Rapa 14 ppm) which was much less pronounced in the restricted diet (30% DR) group (Figure S6b).

Previously, we have developed a method for the indirect detection of DNA lesions in transcribed regions using gene expression analysis (Vermeij, Dollé, et al., 2016). DNA lesions in transcribed regions can obstruct RNA polymerase II, creating transcription stress (Lans et al., 2019). As DNA damage accumulates stochastically, the likelihood that a gene suffers from a lesion blocking transcription is directly proportional to its length, thus leading to a genome‐wide decline of expression of primarily larger genes (Nakazawa et al., 2020; Vermeij, Dollé, et al., 2016). The distribution of expression changes from large and small genes, as inferred from, for example, liver transcriptomic data, has previously been identified as indicator of transcription stress, increasing with age in rodents and humans, elevated by DNA repair deficiency, but largely alleviated by DR (Milanese et al., 2019; Vermeij, Dollé, et al., 2016). Compared to WT animals, *Ercc1* ^∆/−^ mice showed a highly significant bias for large genes to be underrepresented in the category of genes showing increased expression and overrepresented in the class of genes with reduced expression (Figure 5b,c; gray solid versus black dashed lines; see arrows; and Table S3). While this shift in expression of large versus small genes was strongly attenuated by DR (Figure 5b,c; red lines and Table S3), rapamycin treatment failed to alleviate this parameter of age‐related transcription stress in *Ercc1* ^∆/−^ liver (Figure 5b, c; green lines and Table S3).

Together, these data indicate that rapamycin has a minimal effect on the liver transcriptional landscape and does not induce transcription changes triggered by DR that may underlie its beneficial effects in *Ercc1* ^∆/−^ mice (Vermeij, Dollé, et al., 2016) and WT mice (Miller et al., 2014; Unnikrishnan et al., 2020). More importantly, rapamycin does not prevent transcription stress, which is likely an important mechanism that underlies the anti‐aging effect of DR (Vermeij, Dollé, et al., 2016).

## 3 DISCUSSION

**FIGURE 6:**
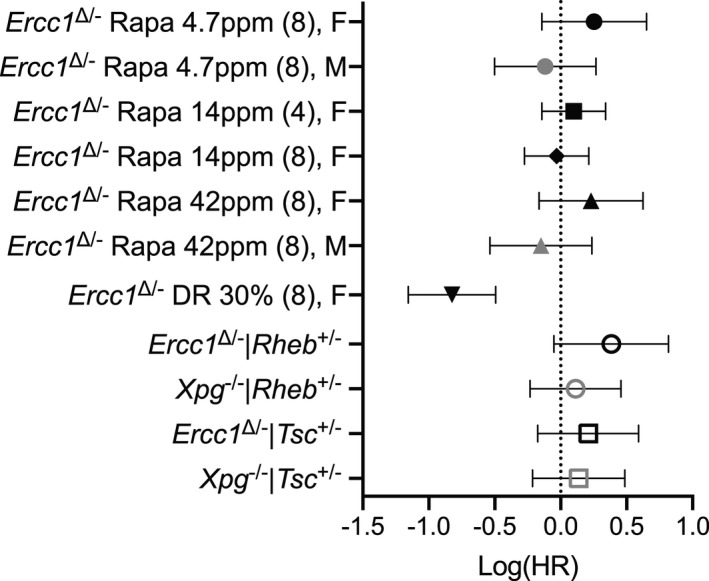
Forest plot of effect size for the logarithm of the hazard ratio (HR; with 95% confidence interval as error bars) for changes in survival of all cohorts represented in this study (data from Figures 1, 2, and 4). Rapamycin treatments per gender were compared to its own *Ercc1* ^∆/−^ control group. Dosage of rapamycin, age of initiation (between brackets), and gender F (females), M (males) are indicated. Genetic modulation of mTOR activity (by *Rheb* ^+/−^ and *Tsc* ^+/−^ mutation) was assessed mixed gender and compared to the appropriate *Ercc1* ^∆/−^ or *Xpg* ^−/−^ controls. Mice subjected to 30% dietary restriction (DR) were compared to the *Ad libitum* mock *Ercc1* ^∆/−^ control from Figure 1h. The direct comparison of DR versus rapamycin yielded a log(HR) of −0.8386 (with 95% CI of −0.5130 to −1.1641)

The quest for DR mimetics has promoted rapamycin as a strong candidate drug for anti‐aging interventions (Blagosklonny, 2019; Fontana et al., 2010; Harrison et al., 2009; Ingram & Roth, 2015). Remarkably, we found that rapamycin did not increase life span of *Ercc1* ^∆/−^ mice, a progeroid DNA repair deficiency mouse model that mimics numerous aspects of aging and responds well to DR (Vermeij, Dollé, et al., 2016). Unlike DR, rapamycin failed to improve important parameters of healthspan: liver, vascular and nervous system pathology, and neurological deficits. We tested rapamycin in multiple cohorts, consistently finding no effect on survival independent of gender, dose, and starting time of the treatment (Figure 6). Importantly, rapamycin treatment resulted in mild weight loss, at the highest dosage of 42 ppm, or at the intermediate dosage of 14 ppm when intervention was started at the early age of 4 weeks, without affecting food intake, indicating that rapamycin in *Ercc1* ^∆/−^ mice at least in part reproduces a main biological effect found in WT mice (Miller et al., 2014). In addition, consistent with mTORC1 inhibition, we found reduced phosphorylation of ribosomal protein S6, a major target of mTORC1, in liver and brain tissue. Finally, we used a genetic approach, which demonstrated that chronic alteration of mTORC1 activity did not have an effect on the life span of *Ercc1* ^∆/−^ or *Xpg* ^−/−^ mice, distinct progeroid mouse models carrying deficiencies in different DNA repair genes, ruling out that inability of rapamycin to extend life span is somehow *Ercc1*‐specific (Figure 6). As all interventions, via different dosages of rapamycin and by genetic means, did not yield health‐ or life span benefits, it would be unlikely that negative and positive effects would cancel out under all these conditions and for all studied endpoints.

Together, this indicates that inhibition of mTOR signaling, including reduced phosphorylation of ribosomal protein S6, fails to improve life span, and does not mediate the beneficial effects of DR in these mice. Even less than 25% of the life span extension exerted by 30% DR would have been easily picked up in our model, stressing the disparity between rapamycin and DR to delay the aging features accelerated in progeroid DNA repair mutants. This points to fundamental differences in the way mTORC1 signaling and DR act, consistent with recent findings of dissimilar effects on multiple pathways and molecular processes, by DR and rapamycin in WT mice, indicating that they extend life span through different mechanisms, again questioning the extent to which rapamycin can be regarded as DR mimetic (Garratt et al., 2016; Thompson et al., 2018; Tyshkovskiy et al., 2019; Unnikrishnan et al., 2020).

Rapamycin may extend survival mainly via acting only on a narrow subset of aging traits, in particular altered immune function and suppression of cancer (Christy et al., 2015; Ehninger et al., 2014; Hasty et al., 2014; Livi et al., 2013). A scenario where the anti‐aging effect primarily results from its anti‐cancer effect would match with our data, as *Ercc1* ^∆/−^ mice show accelerated aging without development of neoplasia throughout their (brief) life span (Dollé et al., 2011; Marteijn et al., 2014) rendering an anti‐cancer drug ineffective in modifying life span of these progeroid DNA repair‐deficient mice. mTOR was recently shown to be required for the survival of mice with short telomeres (Ferrara‐Romeo et al., 2020). Although the *Ercc1* ^∆/−^ mice age accelerated, they and related progeria patients do not have shortened telomeres (Zhu et al., 2003). Rapamycin has been found to extend life span in progeroid Lamin A/C‐deficient mice that models Hutchinson–Gilford progeria syndrome (HGPS) (Kawakami et al., 2019; Ramos et al., 2012). These mice display overall increased mTORC1 activity, and the beneficial effect has been linked to reversal of this increased mTOR activity. However, unlike Lamin A/C‐deficient progeroid animals, DNA repair‐deficient *Ercc1* ^∆/−^ mice show more dissimilar mTORC1 changes with at least suppressed mTOR activity with concomitant elevated autophagy in brain.

Our transcriptome analysis reveals that rapamycin and DR have effects on the *Ercc1* ^∆/−^ liver transcriptome that are qualitatively and quantitatively very different, with rapamycin‐fed mice clustering with the *ad libitum* fed *Ercc1* ^∆/−^ controls. These data are consistent with transcriptome analyses of liver from WT mice, showing that the limited amount of changes induced by rapamycin hardly overlap with those changed by DR (Tyshkovskiy et al., 2019; Unnikrishnan et al., 2020). This indicates that rapamycin and DR largely work via different mechanisms, DR in part via attenuating the GH‐axis (Tyshkovskiy et al., 2019), whereas rapamycin might work via mechanisms that cannot be easily altered in the *Ercc1* ^∆/−^ mice. Earlier studies have shown that short‐lived *Ercc1* ^∆/−^ and *Xpg* ^−/−^ DNA repair‐deficient mice themselves induce a protective “survival response”, suppressing growth and hormone levels (e.g., GH, IGF1, TH) and boosting anti‐oxidant and maintenance mechanisms (Milanese et al., 2019; Niedernhofer et al., 2006; van der Pluijm et al., 2007) and Table S2), mimicking the response induced by DR and in long‐lived GHR‐KO dwarf mice (Schumacher et al., 2008; Vermeij, Dollé, et al., 2016). Intriguingly, rapamycin also fails in increasing life span in GHR‐KO mice, but rather shortens their life span (Fang et al., 2018). This reduced longevity of GHR‐KO mice has been linked to potential effects of rapamycin such as disrupted glucose and lipid metabolism homeostasis, and reduced immune function (Fang et al., 2018). The data of the GHR‐KO mice indicate that the context of lower GH signaling as observed in our *Ercc1* ^∆/−^ and *Xpg* ^−/−^ mice may mask the beneficial effects of rapamycin.

The central remaining question is as follows: How does DR delay aging in *Ercc1* ^∆/−^ mice? Several interventions in DNA repair‐deficient mice have targeted specific pathways underlying mechanisms connected to DR (Alyodawi et al., 2019; Czerwinska et al., 2018; Fuhrmann‐Stroissnigg et al., 2017; Kim et al., 2020; La Fata et al., 2017; Lavasani et al., 2012; Milanese et al., 2019; Tilstra et al., 2012; van Beek et al., 2017; Vermeij, Dollé, et al., 2016; Wu et al., 2017). However, none of these resulted in life span and healthspan extensions in the range of those by DR, although some benefits have been documented on specific tissues and readouts (Alyodawi et al., 2019; van Beek et al., 2016, 2017; La Fata et al., 2017; van der Lugt et al., 2019). Despite decades of intense research the mechanism(s) underlying DR remain(s) largely unresolved. One possibility is that DR acts through multiple pathways and requires combinatorial interventions, each targeting distinct pathways affected by DR. As explained below, another possibility is that DR lowers endogenous reactive species, reducing DNA damage and transcription stress, which are factors underrepresented in current anti‐aging studies.

Interestingly, rapamycin‐mediated inhibition of mTORC1 has recently been implicated in DNA damage response signaling and transcription in WT and long‐lived mutants (Dominick et al., 2017; Ma et al., 2018; Panday et al., 2017; Xie et al., 2018). As *Ercc1* ^∆/−^ mice are genetically compromised in at least four DNA repair mechanisms, enhancing DNA damage response signaling is expected to fail in extending life span in these mice. This idea is fully in line with our findings; especially the absence of an effect by rapamycin on the number of P53‐positive nuclei (Figure 3g,h), as marker of genotoxic stress in brain, transcription changes of DNA damage response and cell fate markers (Figure S6b) and transcription stress in liver (Figure 5b,c and Table S3), in sharp contrast with DR (Vermeij, Dollé, et al., 2016). Hence, an important anti‐aging effect of DR may be triggering the protective “survival response”, which includes boosting anti‐oxidant defenses and metabolic redesign thereby reducing the DNA damage load, consistent with the alleviation of genome‐wide transcription stress by DR. Since repair‐deficient progeroid mutants are particularly sensitive to DNA damage, this would explain their overresponse to DR. As DNA damage and genome instability are important contributors to aging (Hoeijmakers, 2009; Niedernhofer et al., 2018), determining transcription stress may be a valuable parameter for assessing the potential of DR mimetics in reducing DNA damage.

## 4 EXPERIMENTAL PROCEDURES

### 4.1 Ethics statement

All of the animals used in the experimental procedures were in accordance with the Principles of Laboratory Animal Care and with the guidelines approved by the Animal Ethical Committee of the National Institute for Public Health and the Environment (DEC no. 200900263, 201100026, and 2016‐0047‐011) or Erasmus MC (DEC no. 139‐12‐12 and 139‐12‐13) in full accordance with European legislation (Recommendation 2007‐526‐EC). Animals were maintained in a controlled environment (20–22°C, 12 h light:12 h dark cycle) and were housed in individual ventilated cages under specific pathogen‐free conditions. Animals were group housed at the RIVM location when possible and individually housed at the EMC location. Food and water were offered *ad libitum* unless otherwise stated. All efforts were made to ameliorate the suffering of the animals.

### 4.2 Mouse models

The generation and characterization of *Ercc1* ^∆/+^ and *Ercc1* ^+/−^ mice have been previously described (Weeda et al., 1997). *Ercc1* ^∆/−^ mice were obtained by crossing *Ercc1* ^∆/+^ (in a pure C57BL6J or FVB background) with *Ercc1* ^+/−^ mice (in a pure FVB or C57BL6J background, respectively) to yield *Ercc1* ^∆/−^ offspring with a genetically uniform F1 C57BL6J/FVB hybrid background (see ref. (Barnhoorn et al., 2014) for motivation). Wild‐type F1 littermates were used as controls. Similarly, *Xpg* ^−/−^ mice have been characterized previously (Barnhoorn et al., 2014) and were generated by crossing *Xpg* ^+/−^ (in a pure C57BL6J background) with *Xpg* ^+/−^ mice (in a pure FVB background). *Rheb* ^+/−^ mice and *Tsc* ^+/−^ mice have both been described previously (Goorden et al., 2011) and were of a pure C57BL6J background. These heterozygous lines were first crossed with *Ercc1* ^∆/+^, *Ercc1* ^+/−^, and *Xpg* ^+/−^ mice (all in pure C57BL6J backgrounds) prior to the generation of F1 hybrid *Ercc1* ^∆/−^ and *Xpg* ^−/−^ mice. Hence, all animals used in the studies described here were of the same genetic F1 C57BL6J/FVB hybrid background. Typical unfavorable characteristics, such as blindness in an FVB background or deafness in a C57BL6J background, do not occur in this hybrid background. All experiments were used with male and female mice, with numbers for each indicated, unless otherwise stated. Animals were divided randomly over all groups to prevent selection bias. Mice were clinically diagnosed daily in a blinded manner and, weighed, visually inspected weekly, and where indicated scored in a blinded fashion for gross morphological and motor abnormalities. Since the *Ercc1* ^∆/−^ and *Xpg* ^−/−^ mice were smaller, food was administered within the cages and water bottles with long nozzles were used from around two weeks of age.

### 4.3 Dietary treatment with rapamycin

All animals were bred and maintained on AIN93G synthetic pellets (Research Diet Services B.V.; gross energy content 4.9 kcal/g dry mass, digestible energy 3.97 kcal/g, using 2.3 g/kg choline chloride instead of choline bitartrate). Microencapsulated rapamycin (eRapa) and empty microcapsules (Eudragit S100) were obtained from Southwestern Research Institute (San Antonio, TX, USA). 46.7, 140, and 420 mg eRapa, containing 10% Rapamycin, was added per kg AIN93G food mix, resulting in a 4.7, 14, and 42 ppm rapamycin supplemented diet. For the microcapsule control diet, 378 mg empty microcapsules were added per kg AIN93G food mix. Otherwise, normal AIN93G was used as control. The diets were processed into pellets which were radiated with 9 kGy (Isotron, Ede, the Netherlands). Supplemented eRapa and control diets were supplied *ad libitum* to the mice at either 4 or 8 weeks of age for the remainder of their life. On average, *Ercc1* ^∆/−^ and *Xpg* ^−/−^ mice ate 2.3 g food per day. Dietary restriction was initiated at 7 weeks of age with 10% food reduction (2.1 g/day), when animals reached almost maximum body weight and development was completed. Dietary restriction was increased weekly by 10%, until it reached 30% dietary restriction (1.6 g/day) from 9 weeks of age onward. Food was given to the animals just before the start of the dark (active) period to avoid alteration of the biological clock.

### 4.4 Phenotype scoring

The mice were weighed and visually inspected weekly, and were scored in a blinded manner by three experienced research technicians for the onset of various phenotypical parameters. Whole‐body tremor was scored if mice were trembling for a combined total of at least 10 s when put on a flat surface for 20 s. Impaired balance was determined by observing the mice walking on a flat surface for 20 s. Mice that had difficulties in maintaining an upright orientation during this period were scored as having imbalance. Mice showing an abnormal curvature of the spine were scored as having kyphosis.

### 4.5 Behavioral analyses

Rotarod performance was assessed by measuring the average time spent on an accelerating rotarod (Ugo Basile). All animals were given four consecutive trials of a maximum of 5 min with inter‐trial intervals of 1 h. For weekly monitoring, the motor coordination performance was measured with two consecutive trials of a maximum of 5 min.

### 4.6 Immunoblotting

Liver extracts were prepared by mechanical disruption in lysis buffer (150 mM NaCl, 1% Triton X‐100, 50 mM Tris), which was supplemented with mini complete protease inhibitor (Roche Diagnostics) and phosphatase inhibitors (5 mM NaF, 1 mM Na‐orthovanadate). After mechanical disruption, lysates were incubated on ice for 1 h and subsequently centrifuged at 4°C for 20 min. Lysate (25–50 μg) was loaded on a 10% SDS–PAGE gel (Life Technologies LTD) and transferred to a PVDF transfer membrane (GE‐Healthcare Life Sciences). Rabbit anti‐S6 (Cell Signaling Technology; 2217S Lot5, RRID:AB_331355; 1:2000), rabbit anti‐pS6 (Ser 240/244, Cell Signaling Technology; 2215 Lot 14, RRID:AB_331682; 1:500), and mouse anti‐β‐Actin (Sigma Aldrich; A5441 Lot064M4789V, RRID:AB_476744; 1:25.000) were used for detection, semi‐quantified using the ImageJ software package (http://rsb.info.nih.gov/ij/index.html), and phosphorylated:total ratios relative to *ad libitum* samples were calculated.

Brain extracts were prepared by mechanical disruption in RIPA lysis buffer (50 mM Tris, 150 mM NaCl, 0.5% Sodium Dyoxycholate, 1% NP40, 1% SPS) which was supplemented with mini cOmplete protease inhibitor (Roche Diagnostics) and PhosStop phosphatase inhibitor (Roche Diagnostics). After mechanical disruption, lysates were subsequently centrifuged at 4°C for 20 min and any debris was discarded. Lysates (30 μg) were loaded on a 12 or 15% SDS–PAGE gel and transferred to a Nitrocellulate or PVDF transfer membrane using wet transfer. Membranes were blocked in 5% BSA or 5% milk for an hour at room temperature and incubated overnight at 4 °C. Following primary antibodies were used: rabbit anti‐pS6 (Ser 240/244, Cell Signaling Technology; 5364, RRID:AB_10694233; 1:1000), mouse anti‐S6 (Santa Cruz Biotechnology; SC‐74459, RRID:AB_1129205 1:1000), rabbit anti‐GFAP (DAKO; Z0334, RRID AB_10013382; 1:5000), chicken anti‐Neun (Millipore; ABN78, RRID:AB_10807945; 1:5000), rabbit anti‐Beclin (Cell Signaling Technology; 3738, RRID:AB_490837; 1:1000), rabbit anti‐LC3 (Cell Signaling Technology; 2775, RRID:AB_915950; 1:750), and mouse anti‐Actin (Millipore; MAB1501, RRID:AB_2223041; 1:20.000). Secondary antibodies were either horseradish peroxidase conjugated (HRP; Invitrogen; G‐21234, RRID:AB_2223041; 1:5000) or near‐infrared‐dye conjugated (IRDye; Licor Biotech, 926‐32212/926‐32213/926‐68075/926‐68075/926‐68072/926‐68073; 1:20.000) and were incubated for one hour at room temperature. Antibody recognition was done either with Pierce™ Enhanced Chemiluminescence (ECL) Western Blotting Substate (Thermo Scientific) and Chemi‐Doc MP Imaging system (Biorad) or by Odissey scanner (Licor Biotech). Levels of proteins were quantified using either ImageStudio (Licor Biotech) or ImageLab (Biorad).

### 4.7 FACS analysis of nuclear DNA content

Polyploidy levels were assessed based on propidium iodide (PI) fluorescence using FACS analysis (Duncan et al., 2010; McWhir et al., 1993). A small part of the left lobe (approximately 5 mm^3^) was dissected, cut into small fragments, and suspended in 800 μl PBS using a syringe (21G). A total of 300 μl homogenate was added to 300 μl 100% ethanol for fixation. Samples were stored for at least 24 h before further processing. After fixation, the liver homogenate was washed with ice‐cold PBS and subsequently incubated with a pepsin solution for 20 min. After washing in PBS/Tween‐20, cells were collected in 500 μl PBS supplemented with 5 μg/ml PI and 250 μg/ml RNase and samples were measured using the FACS (FACSCalibur, Becton Dickinson).

### 4.8 Histological procedures

Mice were anaesthetized with pentobarbital and perfused transcardially with 4% paraformaldehyde. The brain specimens were carefully dissected out, post‐fixed for 1 h in 4% paraformaldehyde, cryoprotected, embedded in 12% gelatin, rapidly frozen, and sectioned at 40 μm using a freezing microtome or stored at −80°C until use. Frozen sections were processed free floating using the ABC method (ABC, Vector Laboratories) or single‐, double‐, and triple‐labeling immunofluorescence (de Waard et al., 2010; Vermeij, Dollé, et al., 2016). Primary antibodies (supplier; catalogue number, RRID number; dilution) used in this study were as follows: rabbit anti‐GFAP (DAKO; Z0334, AB_10013382; 1:8000); mouse anti‐NeuN (Millipore; MAB377, AB_2298772; 1:1,000); rabbit anti‐P53 (Leica; NCL‐p53‐CMP5, AB_2744683; 1:1000); rabbit anti‐Calbindin (Swant; CB‐38a, AB_2314070; 1:20,000); mouse anti‐Calbindin (Sigma Aldrich; C9848, AB_2313712; 1:20,000); and goat anti‐FOXP2 (Santa Cruz Biotechnology; sc21069, AB_2107124; 1:1000). Alexa488‐, Cy3‐, and Cy5‐conjugated secondary antibodies raised in donkey (Jackson ImmunoResearch) diluted at 1:200 were used for confocal immunofluorescence. Sections were analyzed and photographed using an Olympus BX40 microscope. Immunofluorescence sections were analyzed using a Zeiss LSM700 confocal microscope. Mean intensities were quantified using Fiji.

### 4.9 Quantitative analysis of Purkinje cells

Quantification of Purkinje cells was done using the Optical Fractionator tool of the StereoInvestigator software package (MBF Bioscience), integrated in a Zeiss LSM700 confocal microscope setup. Cerebellar tissue was coronally cut serially into 40 μm sections. Every 8th section was double‐immunostained for calbindin (Swant; CB‐38a, RRID: AB_2314070; 1:20,000/Sigma Aldrich; C9848, RRID AB_2313712; 1:20,000) that stains the whole Purkinje neuron and FOXP2 (Santa Cruz Biotechnology; sc21069, RRID: AB_2107124; 1:1000) that selectively marks the nucleus of Purkinje cells. For each section, a contour of the cerebellar cortex was drawn in StereoInvestigator and confocal stacks (7–9 section per animal, 10–50 stacks per sections) were systematically sampled within this contour, using 40× oil lens and systematic random sampling (SRS) with a grid sizes of 820 × 520 μm or 740 × 470 μm. Typically, we collected around 275 sampling stacks per animal. In each stack, we counted Purkinje cells on the basis of their FOXP2+ nucleus in a counting box of 150 * 150 * 20 μm. Cells whose nucleus crossed either the upper surface or the lower or right sides of the counting frame were not counted, according to the optical fractionator counting rules. To obtain an estimate of the total number of Purkinje cells, we divided the counted number of cells by the relative fraction of tissue counted using the following formula

$$N=\sum Q^{‐}\frac{t}{h}*\frac{1}{\mathrm{asf}}*\frac{1}{\mathrm{ssf}}$$

where *Q* ^−^: The particles counted, *t*: The section mounted thickness, *h* = Counting frame height, asf: Area sampling fraction, and ssf: Section sampling fraction. Using this approach, in WT‐Control mice (*n* = 7) we obtained an estimate of 227.220 ± 36.542 Purkinje cells, which is consistent with numbers found in other studies (Woodruff‐Pak et al., 2010).

### 4.10 Ex vivo vascular function

The responses of isolated aortic tissue were *ex vivo* measured in small‐wire myograph organ baths containing oxygenated Krebs–Henseleit buffer at 37°C. After preconstriction with 30 nM U46619, relaxation concentration–response curves to acetylcholine followed by 0.1 mM sodium nitroprusside, a nitric oxide donor, were constructed (Durik et al., 2012). To specifically reveal endothelium‐dependent responses, which in *Ercc1* ^∆/−^ and their WT littermates almost exclusively consist of nitric oxide signaling, acetylcholine responses were corrected for the response to sodium nitroprusside.

### 4.11 Microarray‐based transcription stress analysis

Total RNA was extracted using QIAzol lysis Reagent from mouse liver. For increased purity, miRNAeasy Mini Kits (QIAGEN) were used. Addition of wash buffers RPE and RWT (QIAGEN) was done mechanically by using the QIAcube (QIAGEN) via the miRNeasy program, and tissue was stored at −80°C. The concentration of RNA was measured by Nanodrop (Thermo Fisher Scientific). RNA quality was assessed using the 2100 Bio‐Analyzer (Agilent Technologies) following the manufacturer's instructions. The quality of the RNA is expressed as the RNA integrity number (RIN, range 0–10). Samples with a RIN below 8 were excluded from analysis. Hybridization to Affymetrix HT MG‐430 PM Array Plates was performed at the Microarray Department of the University of Amsterdam according to Affymetrix protocols. Quality control and normalization were performed using the pipeline at www.arrayanalysis.org.

The linear model from Limma implemented in R was used to identify the DEGs. Pairwise comparisons for each genotype between *ad libitum* and dietary restriction samples were applied to calculate the fold change (FC), *p* value, and false discovery rate (FDR) for each probe set in the microarray. Cutoff values for a DEG were put at FDR <5% with FC ≥ |1.5|. For all mouse analyses, differentially expressed probe sets were considered as DEGs.

Pathway enrichment analysis was conducted via overrepresentation analysis (ORA). ORA was performed in the interactive pathway analysis (IPA) of complex genomics data software (Ingenuity Systems, Qiagen) by employing a pre‐filtered list of differentially expressed genes. The overrepresented canonical pathways were generated based on information in the Ingenuity Pathways Knowledge Base. A pathway was selected as deregulated when the *p* value in the Fisher test was lower than 0.01. Additionally, IPA transcription factor (TF) analysis was performed to identify the cascade of upstream transcriptional regulators that can explain the observed gene expression changes in the different lists of DEGs. To do this, data stored in the Ingenuity Knowledge Base, with prior information on the expected effects between TF and their target genes, were used. The analysis examines how many known targets of each TF are present in the list of DEGs, and also compares their direction of change to what is expected from the literature, in order to predict likely relevant transcriptional regulators. If the observed direction of change is mostly consistent with a particular activation state of the transcriptional regulator (“activated” or “inhibited”), then a prediction is made about that activation state. For each TF, two statistical measures are computed (overlap *p* value and activation z‐score). The overlap *p* value labels upstream regulators based on significant overlap between dataset genes and known targets regulated by a TF. The activation z‐score is used to infer the likely activation states of upstream regulators based on comparison with a model that assigns random regulation directions. Overlap *p* value lower than 0.05 and z‐score higher than |2| were selected as thresholds to identify a TF as relevant.

Limma was used to identify the DEGs among samples compared with the *a* *d libitum* fed control WT samples. Differentially expressed genes were selected using an FDR of <0.05 and a linear fold change of ±1.5. Next, probe sets in the Affymetrix array with multiple gene annotation were filtered out. BiomaRt was used to retrieve the gene length for the remaining probe sets (32,930 probe sets from 45,142 probe sets in the original microarray). Finally, a relative frequency (kernel density) plot of gene length and probability density for DEG in each comparison was drawn using the density function implemented in R. Kernel density estimates are related to histograms, but with the possibility to smooth and continuity by using a kernel function. The y‐axis represents the density probability for a specific range of values in the *x*‐axis.

All data files have been submitted to the NCBI gene expression omnibus (GEO) under accession number GSE149029.

### 4.12 Statistical analysis

All data with error bars are presented as mean ± standard deviation (SD), and the individual datapoints (dots) are presented in the bar graphs. All analyses were performed using GraphPad Prism version 8.4.2 (GraphPad Software, La Jolla California USA) and R software. *p* values expressed as *p < 0.05; **p < 0.01, ***p < 0.001 were considered to be significant; when indicated as “ns,” comparisons should be considered non‐significant. We used Welch's adjusted t test for testing the difference between two groups (also called unequal variances t test), a modified Student's t test under the assumption of unequal variances. Comparisons between more than two groups were carried out by one‐way ANOVA with Tukey's multiple comparisons test. For FACS analysis, differences between groups were assessed by two‐way ANOVA, with age and diet as fixed factors. For vascular dilatation, differences between groups were assessed by general linear model (GLM) for repeated measures. Statistical differences for the gene length transcription stress analysis were determined using the Wilcoxon–Mann–Whitney (WMW) test.

Weekly body weights from the age of intervention‐start through the maximum age at which all groups of the respective experiment still had ≥80% survival were included, excluding the weights of the animals that died during that period. Subsequently, all the body weights after the start of the intervention were log_10_‐transformed, had the log_10_ values at the start of the intervention subtracted from them, and then, for convenience (this being no more than a normalization without any effect on the statistical tests), had the lowest log_10_‐baseline weight of the entire dataset subtracted from them. Then, the area under the curve (AUC) was determined by the trapezoid rule for the entire selected time interval for each animal. Grubbs’ test in GraphPad Prism, with alpha = 0.01, was applied once to all AUC values of the experiment to identify and remove potential outliers. Mean AUCs between groups were compared by Welch's *t* test.

Statistics for life span and onset of phenotypes were performed by computing Kaplan–Meier estimates of the survival curves and estimates of the log hazard ratio effect size with 95% confidence intervals, and comparing the survival functions of different groups with the log‐rank test. These analyses were carried out in GraphPad Prism and in R with the packages “survival” and “coin.” When comparing two groups consisting of both female and male mice, the log‐rank test was additionally done by stratifying (blocking) on sex.

## CONFLICT OF INTEREST

The authors declare no competing interests.

## AUTHOR CONTRIBUTIONS

J.H.J.H., W.P.V., H.v.S., and M.E.T.D. performed conceptualization. M.B.B., D.J., W.P.V., and M.E.T.D. designed and wrote the manuscript. All authors contributed to editing the manuscript. R.M.C.B., S.B., N.v.V., S.I., C.T.v.O., B.N., W.P.V., and M.E.T.D. performed and analyzed the mouse cohorts. S.I. performed FACS analysis of nuclei and S6 related western blotting. E.P.F. and A.J.M.R. quantified vascular function. D.J., R.M.C.B., S.B., N.v.V., and W.P.V. performed and analyzed phenotypical scoring and behavioral analysis. M.B.B. and D.J. characterized neuropathological changes. J.L.A.P. and W.P.V. performed transcriptomic analyses. J.A.F and W.P.V. performed statistical analyses with body weight and life span data. R.M.C.B. and Y.E. performed the genetic interventions. D.J., J.H.J.H., W.P.V., and M.E.T.D. carried out supervision.

### Open Research Badges

This article has been awarded <Open Data, Open Materials> Badges. All materials and data are publicly accessible via the Open Science Framework at https://www.ncbi.nlm.nih.gov/geo/query/acc.cgi?acc=GSE149029.

## Supporting information

Fig S1

Fig S2

Fig S3

Fig S4

Fig S5

Fig S6

Table S1

Table S2

Table S3

## Data Availability

All data generated or analyzed during this study are included in this published article (and its supplementary information files).
